# Seasonal Variation of Harbor Seal's Diet from the Wadden Sea in Relation to Prey Availability

**DOI:** 10.1371/journal.pone.0155727

**Published:** 2016-05-13

**Authors:** Camille de la Vega, Benoit Lebreton, Ursula Siebert, Gael Guillou, Krishna Das, Ragnhild Asmus, Harald Asmus

**Affiliations:** 1 AlfredWegener-Institut Helmholtz-Zentrum für Polar- und Meeresforschung, Wattenmeerstation Sylt Hafenstr. 43, D-25992 List/ Sylt, Germany; 2 Littoral Environnement et Sociétés (LIENSs), CNRS-Université de la Rochelle, Bâtiment ILE 2, rue Olympe de Gouges 17 000 La Rochelle, France; 3 Stiftung Tierärztliche Hochschule Hannover Institut für Terrestrische und Aquatische Wildtierforschung Werftstr. 6, 25761 Büsum, Germany; 4 Laboratory of oceanology—MARE center, Université de Liège, Allée de la Chimie 3, Bât. B6c, 4000 Liège (Sart-Tilman), Belgium; University of Connecticut, UNITED STATES

## Abstract

The Wadden Sea has an important role for marine mammals in terms of resting, nursing and foraging. Harbor seal is the most abundant marine mammal species in this area. The use of the food resources of the Wadden Sea by seals is not clear, and previous studies showed that this species can travel kilometers away from their haul-outs to forage in the North Sea. In this study, we analyzed the stable isotopes of vibrissae from 23 dead harbor seals found on the island of Sylt to investigate their diet. The predator´s carbon and nitrogen isotope compositions were compared to the compositions of different potential prey items from the Sylt-Rømø Bight and from the North Sea in order to study seasonal pattern in the diet and in the foraging location. In parallel, seasonal variation of abundance and biomass of the potential prey items from the Sylt-Rømø Bight were studied and compare to their contribution to the seal´s diet. The results revealed a change in the seal´s diet from pelagic sources in spring to a benthic based diet in summer, and an increasing use of the North Sea resources in fall and winter in accordance with the seasonal variation of the availability of prey in the Sylt-Rømø Bight.

## Introduction

Marine mammals represent the most prominent members among top predators in the marine environment [1]. Their abundance and distribution can have a large effect on the structure and the functioning of ecosystems and communities [2–4]. Assessing the role of top predators in the functioning of ecosystems is then a central issue in ecology and management [4]. Nevertheless, the role of top predators in structuring ecosystems is still not well known [4, 5] due to their ecological niches often exceeding the temporal and spatial scales which are used to define community boundaries [5, 6].

In the Wadden Sea, harbor seal (*Phoca vitulina*) is, together with harbor porpoise (*Phocoena phocoena*), the most abundant marine mammal species [7]. The conservation measures introduced in the 1970s for marine mammals [1, 8–10], and particularly the protection of harbor seals by the hunting prohibition started in 1976 for the entire Wadden Sea [11], allowed its population to grow [1, 9, 11]. Despite two epizootics in 1988 and 2002 which interrupted the upward trend in population growth sharply [7], the Wadden Sea population of harbor seals increases and might approach the carrying capacity of the area [12, 13], with 26 576 individuals counted on land in August 2014 [14]. Harbor seals´ population spreads from Denmark to the Netherlands, with ~61% of its population located along the German coast [14]. The Wadden Sea is an important habitat for harbor seals in terms of reproduction [7, 14, 15] and food resources [8]. Harbor seals use the numerous sand banks regularly exposed at low tide in different bays of the Wadden Sea to give birth, rest and molt [16]. They also use the Wadden Sea at high tide to forage on the abundant food stock it provides [12].

Harbor seals are opportunistic feeders subsisting largely on fish, although mollusks and crustaceans may sometimes form a significant part of their diet [17, 18]. Several studies based on seal stomach contents conducted in the North Sea showed a variation in the dominant species in the seal´s diet depending on the location, the main prey species being either gadoids and flat fish [19–22], or clupeids and sand eels [23–26]. Along the German coast, in the Schleswig-Holstein area, gadoids (G*adus morhua* and *Merlangius merlangus*) and flat fish (*Limanda limanda*, *Platichthys flesus* and *Pleuronectes platessa*) are prominent in the seal´s diet with *Ammodytes tobianus* and *Clupea harengus* of secondary importance [17, 18, 27]. Thus, harbor seals feed on a wide range of prey with the prevalence of some key species. The contributions of these prey items to the diet vary depending on the area, and likely depending on the prey availability [25, 28].

Due to their large body size and their high abundance in the Wadden Sea, seals exert a strong pressure of predation on their environment [4, 12]. Even if harbor seals from the Wadden Sea appear to use the North Sea more than previously expected [6, 29], they might exert a pressure of predation on the Wadden Sea food resources. Consequently, there are needs to improve the understanding of the trophic behavior of seals in the North Sea and in the Wadden Sea, in order to have better estimations of their diets and to determine spatio-temporal variations of their foraging activities. This would allow evaluating their influence on the ecosystems in which they live, in order to improve management plans for conservation of seals and of their food resources.

Stable isotope analysis is a powerful tool for determination of food resources used by marine mammals [5, 30–32]. This method is very complementary to gut content analyses, which have already been carried out on seals from the same area [17, 18, 27]. Gut content and feces analyses give a snapshot of the ingested prey items whereas the stable isotope composition provides dietary information integrated over few days (e.g., plasma, liver) to few months (e.g., muscle, hair) in function of the differences of metabolic activity (e.g., turnover) or growth rate between the tissues [33]. The stable isotope composition of carbon in predator tissue reflects the origin of food resources: it allows generally a good discrimination between food resources produced in continental areas, those produced in the open ocean and the ones produced in benthic environments [34–36]. The stable isotope composition of nitrogen is commonly used as an indicator of the trophic position of a consumer, thanks to the large trophic fractionation observed for nitrogen between each trophic level [37–39]. For the present study, stable isotope analyses were carried out on vibrissae to determine temporal patterns of diet. Indeed, due to daily growth of vibrissae and their metabolic inertia [40], their isotopic composition reflects the diet at the time of their growth [41]. Several studies revealed that vibrissae provide a powerful way to assess diet and foraging location of marine mammals such as elephant seals (*Mirounga leonine)* [42], leopard seals (*Hydrurga leptonyx*) [40], harp seals (*Pagophilus groenlandicus*) [43] and sea otters (*Enhydra lutris nereis*) [44]. Zhao and Schell (45) showed that harbor seal´s vibrissae can archive ecological changes over a long metabolic period. As a result and knowing their growth rate (0.78 mm.d^-1^ from May to September and 0.075 mm.d^-1^ from October to April) [45] vibrissae segmental isotopic analysis provides an efficient tool for studying foraging ecology of harbor seals giving precise (day) and long term (up to one year) information about the history of their food resources.

The present study aims to first estimate the temporal variation of the diet of harbor seals from the German Wadden Sea using stable isotope analyses, focusing both on the different type of prey items (i.e., trophic groups of prey species) and the different origins of these prey items (North Sea *vs*. Sylt-Rømø Bight). The probability to be part of the seal´s diet is then related to the seasonal patterns of the prey species´ biomass and abundance.

## Material and Methods

### Ethic Statement

In the Wadden Sea area, harbor seals are protected under the Annex II of the Convention on Migratory Species of Wild Animals, also called Bonn Convention [46], and particularly since 1991 under the protection of the Trilateral Seal Agreement between Denmark, Germany and the Netherlands (Agreement on the Conservation of seals in the Wadden Sea, Bonn Convention) [47]. In addition, they are protected under Annex III (protected fauna species) of the Convention on the conservation of European wildlife and natural habitats (Bern Convention, 1985) [48] The harbor seal is also listed in the Annexes II and V of the EU Habitats Directive (consolidated version 2007) [49] on the conservation of natural habitats and of wild fauna and flora. Harbor seals are classified with least concern in the regional red list for Germany (Federal Agency for Nature Conservation 2009) [50] and in the European red list (IUCN 2012) [51]. All seal samples were taken in accordance with these protection measures. Samples were collected as part of a harbor seals stranded network, established on the German coast of Schleswig-Holstein after the 1988/1989 Phocine Distemper Virus epidemic [52]. All stranded seals were found dead or were killed because of serious illness by authorized seal hunters affiliated to the authorities of Schleswig-Holstein Wadden Sea National Park.

The sampling of prey species from the Sylt-Rømø Bight were part of a monthly fish monitoring supervised by the Alfred Wegener Institute since 2006. No endangered prey species were used in this study. All caught fish, squid and shrimp individuals were measured (length and weight) on board as fast as feasible for biomass and abundance survey, and have been returned to the wild after being held in water. The individuals sampled for stable isotope analyses were rapidly killed and stored in a freezer on board. The individuals of prey species from the North Sea were collected for stable isotope analyses among catches of a professional shrimp trawler from the island of Rømø.

### Study site

**Fig 1:**
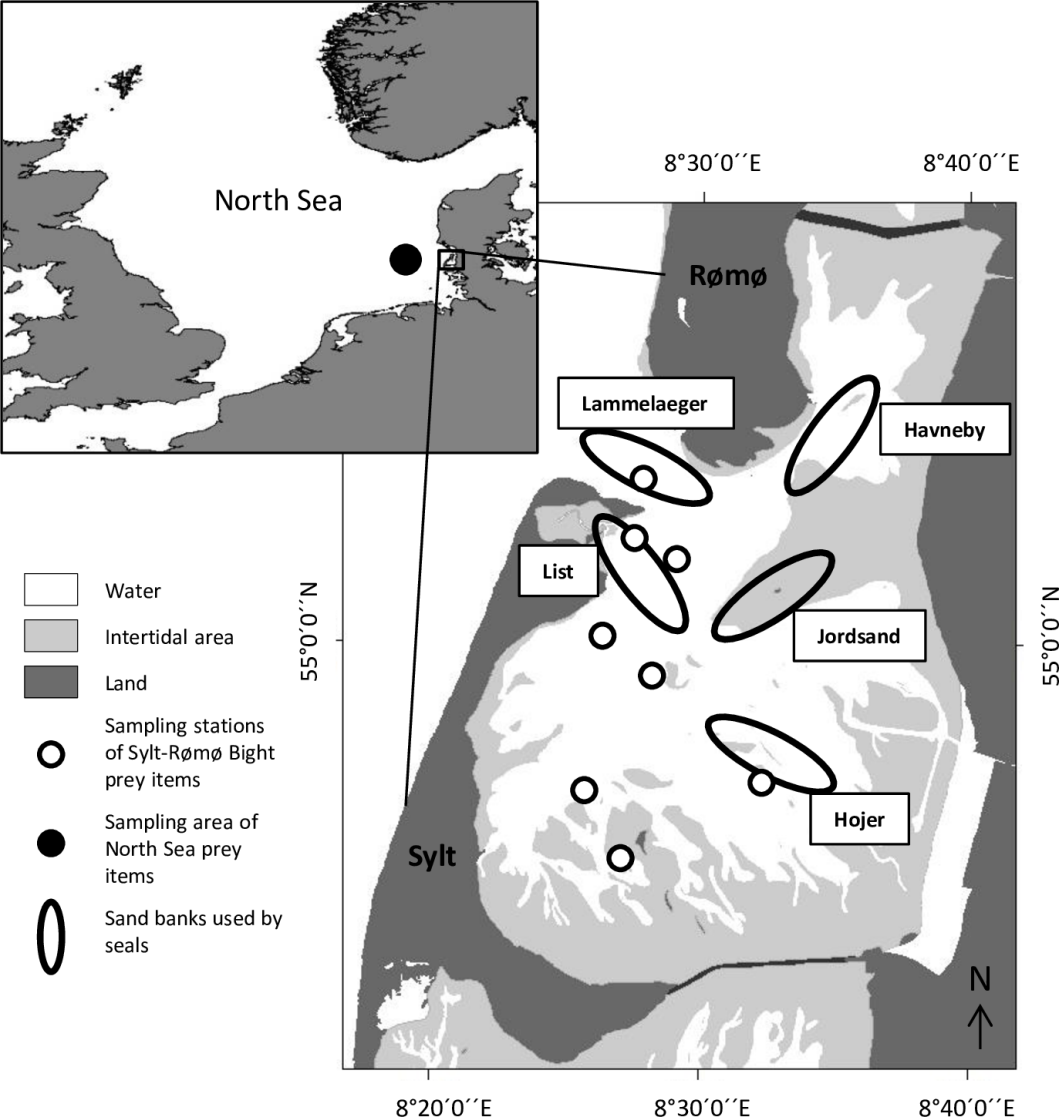
Location and map of the Sylt-Rømø Bight; Maps created using ArcGIS® 10 Esri software. Sylt-Rømø Bight map data courtesy of the Schleswig-Holstein’s Government-Owned Company for Coastal Protection, National Parks and Ocean Protection—National Park Authority, Tönning.

The Sylt-Rømø Bight (54°52’–55°10’ N, 8°20’–8°40’ E) is part of the Wadden Sea, which extends along the south-eastern margin of the North Sea from the Netherlands to Denmark. This 404 km^2^ semi-enclosed basin is located between the islands of Sylt (Germany) and Rømø (Denmark; Fig 1). Two causeways connect the islands with the mainland, and prohibit any exchange of water with the adjacent tidal basins. The only connection to the North Sea is a deep tidal channel between the two islands. The tidal range inside the Bight is up to 2 m [53]. The Sylt-Rømø Bight provides shelter for a stable colony of ≈ 470 ± 97 harbor seals on average in summer (2009 to 2012) [54]. Harbor seals use five sand banks uncovered at low tide as haul out sites. These sandbanks are spread in the whole Bight, with the Jordsand and List sand banks (Fig 1) being the most frequented [54].

### Prey samples

Fish biomass and stable isotope samples were measured and collected from the catches of fish monitored monthly from 2008 to 2013 in the Sylt-Rømø Bight (Fig 1).

#### Sampling for fish biomass and abundance

Sampling for biomass and abundance of the prey species took place monthly from 2010 to 2012 at eight stations in the Sylt-Rømø Bight (Fig 1) to provide a representative geographical coverage of the area. Two hauls were carried out at every station: one in the water column and one at the bottom, each for 15 minutes at an average speed of approximately 1 m.s^-1^. Sampling was carried out using a 17 m long mini bottom trawl, also designed to be deployed for pelagic fishing. The mouth of the net was up to 7 m in width and 3 m in height. Mesh size measured 32 mm in the wings, 16 mm in the mid part and 6 mm in the cod end. Fish, shrimps and squids were identified to the species level, measured to the nearest 0.5cm and counted. Fish biomass was estimated using the following length-weight relationship: *WW* = *a* × *l*^*b*^, with *WW*: wet weight in g, *l*: length in cm, and *a* and *b*: constants calculated by Pockberger [55] for every species sampled during the fish monitoring in the Sylt-Rømø Bight. Catch per unit of effort (CPUE), i.e., the number (CPUE_n_) or biomass (CPUE_m_) of fish caught per hour of sampling, was calculated using the following equations: *CPUE*_*n*_ = ∑*n*/*t* and *CPUE*_*m*_ = ∑*m*/*t*, with *n*: number of individuals, *m*: biomass of individuals (g) and *t*: fishing time (hour). The number of individuals was summed by group of prey items (see section 2.5 Data and statistical analyses).

#### Sampling for stable isotopes of prey

Potential prey species of harbor seals (i.e., fish, shrimps and squids) were sampled in the Sylt-Rømø Bight and in the North Sea in order to determine their difference in stable isotope composition between these two areas. Potential prey species from the Sylt-Rømø Bight were sampled seasonally from April 2008 to November 2009 [56] and from January to November 2013. Potential prey species from the North Sea were collected from May to September 2013 by a professional shrimp trawler. The opening size of the net was 5 meters and mesh size was 20 mm. Three individuals from the most abundant size-class of each species were collected, measured to nearest 0.5 cm and then stored at -20°C for further analysis.

### Sampling for stable isotopes of seals

Twenty three harbor seal carcasses in good state of conservation were collected from June 2012 to February 2014 along the coastline of the Sylt Island. This sampling represents about 5% of the population of harbor seals in the Sylt-Rømø Bight during summer (470 individuals on average) and encompasses the totality of stranded adults and most of the stranded young-of-the-year older than 3–4 months collected by the seal´s hunter on the Sylt coast during this period. Necropsies were conducted on the carcasses at the Institute for Terrestrial and Aquatic Wildlife Research (ITAW) of University of Veterinary Medicine Hannover Foundation, according to the protocol described by Siebert, Wohlsein [57]. Until necropsy, the carcasses were stored frozen in plastic bags at -20°C. The age (older than 2 years *vs*. young-of-the-year) was estimated according to the length. The estimated age of the young-of-the-year (in months) was determined as the number of months between the main birth period (May to June) [15] and the day of collection (Table 1).

**Table 1:** Sex, finding date and age of the twenty three sampled harbor seals.

seal ID	sex	finding date	age
1	m	29-Jul-12	13–14 months
2	m	8-Sep-12	> 2 year
3	m	21-Sep-12	> 2 year
4	f	30-Sep-12	3–4 months
5	m	30-Sep-12	3–4 months
6	m	7-Oct-12	4–5 months
7	f	10-Oct-12	4–5 months
8	m	13-Oct-12	4–5 months
9	m	19-Oct-12	4–5 months
10	m	6-Dec-12	6–7 months
11	f	9-Dec-12	6–7 months
12	f	9-Dec-12	6–7 months
13	f	10-Dec-12	6–7 months
14	f	31-Dec-12	6–7 months
15	f	31-Dec-12	6–7 months
16	f	31-Dec-12	6–7 months
17	f	24-Mar-13	9–10 months
18	m	29-Mar-13	9–10 months
19	f	24-Apr-13	> 2 year
20	m	12-Jul-13	> 2 year
21	m	13-Nov-13	5–6 months
22	m	13-Nov-13	5–6 months
23	f	11-Feb-14	> 2 year

To evaluate the similarity between vibrissae originating from the same animal, two different vibrissae were collected on seals #1 (adult) and #4 (yearling) (i.e., four vibrissae in total). The R² of the linear regression between the two vibrissae from a same individual were calculated to verify the similarity between stable isotope compositions and growth rate. We observed a very good similarity between 2 vibrissae from a same individual for both δ^13^C and δ^15^N values (δ^13^C: seal #13: R^2^ = 0.804, seal #29: R^2^ = 0.975; δ^15^N: seal #13: R^2^ = 0.991, seal #29: R^2^ = 0.944; S1 Fig). The longest mystacial vibrissae were sampled for each individual in order to cover the longest period of growth.

### Preparation and analysis of stable isotope samples

Prey samples were freeze-dried and ground individually to a fine powder using a ball mill. Whole eviscerated individuals were analyzed. To avoid the bias due to presence of CaCO_3_ from fish bones, samples for δ^13^C analyses were acidified using 1 mol.L^-1^ hydrochloric acid, then dried at 60°C and ground again [58, 59]. δ^15^N analyses were carried out on raw samples in order to avoid any potential bias due to acidification.

Harbor seal vibrissae were cleaned using soap in an ultrasonic bath for 10 minutes and then rinsed 4 times in distilled water. Vibrissae were measured, dried and sliced with a sharp cutter in 1 to 2 mm consecutive sections (ranging in mass from 0.8 to 1.5 mg) starting from the proximal end [41]. This represented a trade-off between the number of sections (and hence the temporal resolution attainable for the isotopic time series) and the size of the sample [42]. The number of samples analyzed per vibrissae varied from 18 to 42 depending on its length.

Each piece of vibrissae and homogenized powdered samples of prey were precisely weighed (± 1 μg) and were sealed in a tin capsule for stable isotope analyses. Samples were processed on an elemental analyzer (Vario Microcube, Elementar, Germany) coupled to an isotope ratio mass spectrometer (Isoprime 100, Isoprime, UK). Results are expressed in the δ notation as deviation from international standards (Vienna Pee Dee Belemnite for δ^13^C and N_2_ in air for δ^15^N) following the formula: δ^13^C or δ^15^N = [(R_sample_/R_standard_) − 1] x 10^3^, where R is ^13^C/^12^C or ^15^N/^14^N isotopic ratios. Calibration was performed using certified reference materials (IAEA-C6, IAEA-N2, for nitrogen). Analytical precision based on repeated analyses of glycine (p.a. Merck, Germany) used as an internal standard was <0.15‰ for carbon and nitrogen.

### Data and statistical analyses

#### Trophic group of prey items

Fish prey species were grouped following three trophic groups (Table 2): planktivorous/piscivorous, benthivorous/piscivorous and benthivorous, as described in Froese and Pauly (60). The planktivorous/piscivorous group is represented by pelagic species (e.g., *C*. *harengus*, *A*. *tobianus*) living in the water column and feeding on zoo- and phyto-plankton and/or small fishes. The benthivorous/piscivorous group comprises benthopelagic species (e.g., *M*. *merlangus*, *L*. *limanda*, *Myoxocephalus scorpius*) living partly in the water column but foraging on the seafloor. These species are feeding on crustacean, mollusks and polychaetes, but also on small fishes and cephalopods [60]. The benthivorous group consists mainly of demersal species (e.g., *Pomatoschistus minutus*, *P*. *platessa*) living on the seafloor and feeding on small crustaceans, mollusks, polychaetes, fish eggs [60] and, for some groups, on amphipods [61]. Due to its anadromous behavior, *Osmerus eperlanus* was treated separately than the benthivorous/piscivorous group, although it feeds on shrimps, small crustaceans and small fishes [60]. Only squid species belonging to the genus *Loligo* were found.

**Table 2:** Groups of species used as prey items in the Sylt-Rømø Bight (for biomass and stable isotope analyses) and in the North Sea (for stable isotope analyses).

Planktivorous/piscivorous	Benthivorous/piscivorous	Strictly benthivorous
*Ammodytes tobianus*	*Ciliata mustela* ***	*Agonus cataphractus*
*Atherina presbyta* *	*Gadus morhua* ***	*Crangon crangon*
*Belone belone*	*Gasterosteus aculeatus* **	*Pleuronectes platessa*
*Clupea harengus*	*Limanda limanda*	*Pholis gunnellus* **
*Cyclopterus lumpus* *	*Merlangius merlangus*	*Pomatoschistus microps*
*Hyperoplus lanceolatus*	*Myoxocephalus scorpius* **	*Pomatoschistus minutus*
*Scomber scomber* *	*Platichthys flesus* **	*Solea solea*
*Sprattus sprattus*	*Spinachia spinachia* **	*Zoarces viviparus*
*Trachurus trachurus* *	*Syngnathus rostellatus* **	

* species not sampled for stable isotope analysis.

** species only sampled in the Sylt-Rømø Bight for stable isotope analysis.

*** species only sampled in the North Sea for stable isotope analysis.

The seasonal biomass and abundance of trophic groups were similar between the years 2010, 2011 and 2012 (Kruskal Wallis rank sum test: Planktivorous/Piscivorous, all p-values > 0.11 for biomass and > 0.10 for abundance; Benthivorous/Piscivorous, all p-values between > 0.33 for biomass and > 0.26 for abundance; Benthivorous group, all p-values > 0.13 for biomass and all p-values > 0.10 for abundance). Therefore, the seasonal biomass and abundance of groups of prey items were averaged per year in order to have a more robust data set representing the seasonal availability of prey for harbor seals.

The stable isotope compositions of trophic groups from the Sylt-Rømø Bight were similar between years of sampling among seasons (Kruskal Wallis rank sum test for δ^13^C: Planktivorous/Piscivorous, all p-values > 0.19; benthivorous/piscivorous, all p-values > 0.40; Benthivorous group, all p-values > 0.15; *O*. *eperlanus*, p-value > 0.05; Kruskal Wallis rank sum test for δ^15^N: Planktivorous/Piscivorous, all p-values > 0.62; benthivorous/piscivorous, all p-values > 0.08; Benthivorous group, all p-values > 0.05; *O*. *eperlanus*, p-value > 0.70). As a result, the stable isotope compositions of the different trophic groups were averaged on a seasonal basis for the construction of seasonal mixing models (see section 2.7. mixing models).

#### Trophic fractionation factors

δ^13^C and δ^15^N values are expressed as means, generally followed by standard deviations. As a net result of isotopic discrimination (i.e., the differential behavior of the stable isotopes during biochemical or physico-chemical reaction), the stable isotopic composition of a consumer is generally different than those of its potential prey. Such difference, called trophic fractionation factor (TFF) is the net result of all fractionations occurring during metabolism and enrichment is generally observed in heavier isotopes of consumer tissues compared to those of its preys. Isotopic composition of prey and predators was compared considering the trophic fractionation factor values in vibrissae from Hobson, Schell (43): TFF δ^13^C = 3.2‰ and TFF δ^15^N = 2.8‰. Little is known about the variability of TFFs among tissue, species and individuals for marine mammals. For this study, we used 0.8‰ and 0.1‰ as standard deviation for the TFFs of δ^13^C and δ^15^N, respectively, in vibrissae as described in Lesage [62] for hairs, a keratin tissue comparable to vibrissae.

#### Temporal reconstruction of vibrissae

Growth rates used for reconstruction of the temporal variation in stable isotope composition of vibrissae were 0.78 mm.d^-1^ from May to September, and 0.075 mm.d^-1^ from October to April [45]. Most of the harbor seals were still alive when beaching, therefore the day of collection on the beach was considered to be the last day of vibrissae growth. Most of the sampled seals were emaciated and therefore probably starving in the last days of their life. However due to the inertia of this tissue [40], once grown, the stable isotope composition of vibrissae is not modified with time [41, 45]. Nevertheless, to avoid potential bias due to particular feeding behavior or fasting before the death, we removed the sections of vibrissae of potentially starving animals corresponding to the last days of their life from the data set. We thus considered that the vibrissae sections used in this study reflected the stable isotope composition of normally feeding individuals.

In order to exclude the potential influence of lactation and post weaning fast on the stable isotope composition of the young-of-the-year [63–67], we examined the monthly evolution of the δ^15^N and δ^13^C values of vibrissae sections of young-of-the-year and adults from May (i.e., month of birth of young-of-the-year) to December (S1 Table). Both δ^15^N and δ^13^C values of vibrissae sections corresponding to young-of-the-year older than 2–3 months were similar to those of adults (Wilcoxon test, all p-values > 0.1, S1 Table). Therefore, the sections of the vibrissae of young-of-the-year corresponding to months before September were removed for data analyses and sections of the vibrissae of young-of-the-year corresponding to months from and after September were kept for the analyses in order to use only young-of-the-year´s vibrissae reflecting the same stable isotope composition as adults.

The temporal moving mean of δ^13^C and δ^15^N values, taking in account all vibrissae data corresponding to 15 days on either side of the central value (30 days in total), was calculated in order to smooth out the short term and inter-individual variability of isotopic composition, and highlight the monthly trends. An example of the data treatment of 4 vibrissae is detailed in S2 Fig. Seasonal variation of isotopic composition covering the period from March 2012 to February 2014 was divided into the four following intervals and then studied. Spring: March to May (n = 4), summer: June to August (n = 5), fall: September to November (n = 16) and winter: December to February (n = 9).

#### Statistical analyses

Non-parametric procedures were used to achieve more robust statistics in case of non-independence of data within series (e.g., two seasons along the same vibrissae) or small sample size (sample size ≤ 10). Kruskal–Wallis tests were applied on isotopic data in order to compare the different groups of prey items and to test for seasonal isotopic variations. These tests were followed by multiple pairwise comparisons using the Wilcoxon rank sum test. When data were independent and sample size was ≥ 10 (prey items from the Sylt-Rømø Bight), ANOVA followed by Tukey HSD tests were applied.

### Mixing models

Relative contributions of the different prey trophic groups (isotopic sources) from the Sylt-Rømø Bight and from the North Sea, to the harbor seal diet were estimated by running the SIAR (Stable Isotope Analysis in R) mixing model [68] using δ^13^C and δ^15^N values. In the model, individual harbor seal isotope ratios were used while for prey species, means and standard deviations were entered. Trophic fractionation factor values were 3.2 ± 0.8‰ for δ^13^C and 2.8 ± 0.1‰ for δ^15^N.

Four seasonal mixing models (i.e., spring, summer, fall, winter) were built to study seasonal changes of harbor seals food resources. These models were built using the seasonal mean isotopic values of each vibrissa as predator values (spring: n = 4, summer: n = 5, fall: n = 16, winter: n = 9), and the isotopic values per season of the different groups of prey items. For prey items, the yearly average was used when sample size was too small (n < 3; i.e., benthivorous/piscivorous group in winter and *O*. *eperlanus* in summer for the Sylt-Rømø Bight; planktivorous/piscivorous, benthivorous/piscivorous and benthivorous groups in spring and winter for the North Sea).

The models were run for 500 000 iterations and the first 50 000 iterations were discarded. Credibility intervals (CI) of 0.95, 0.75 and 0.25 were computed. CI is a contiguous interval that contains a specified proportion of the posterior probability [69]. For example, if the upper 0.95 CI is A and the lower 0.95 CI is B, the contribution value has 95% chance of lying between A and B.

## Results

### Seasonal variation of the fish biomass and abundance in the Sylt-Rømø Bight

**Fig 2:**
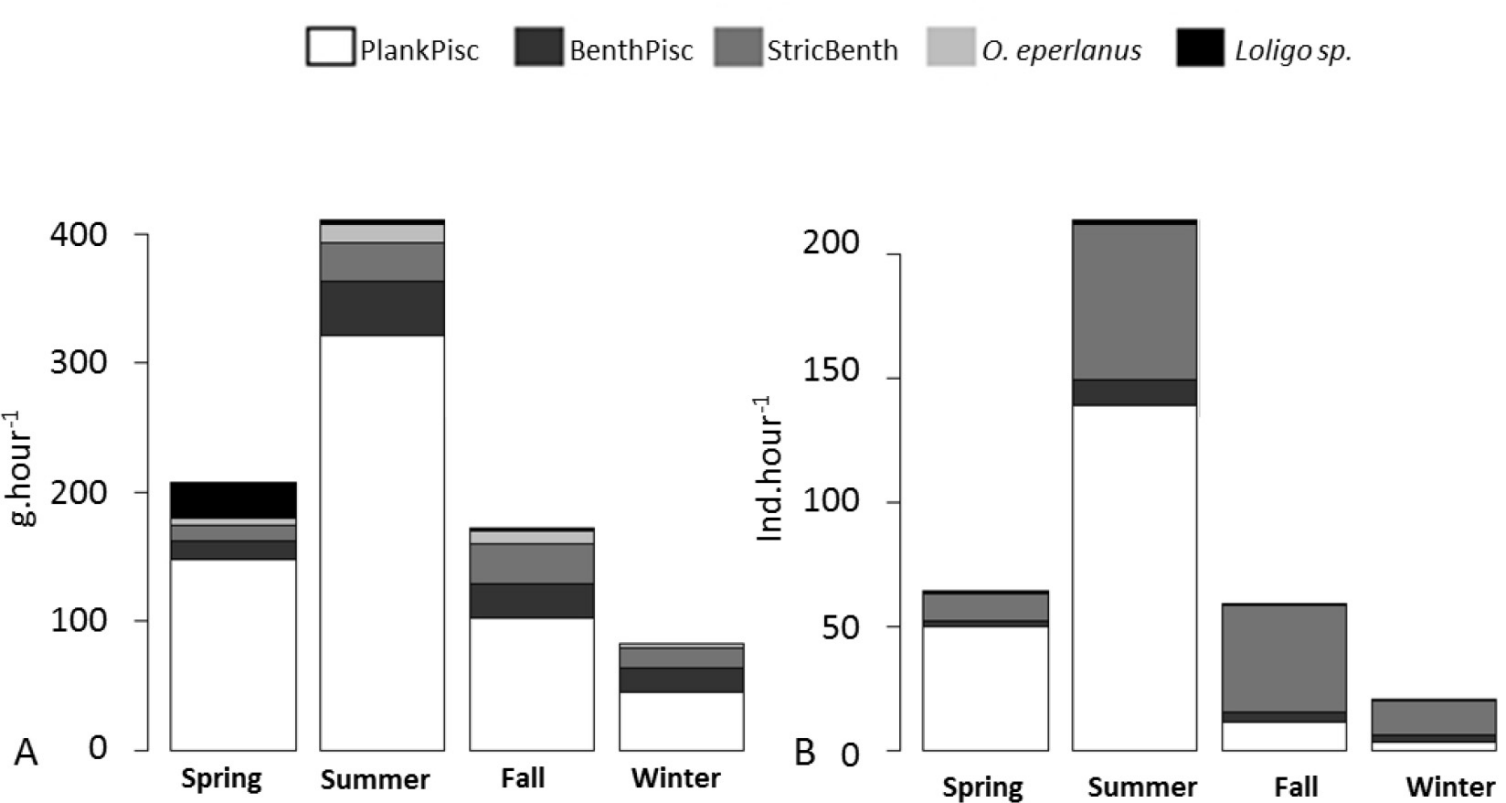
**CPUE_m_ (A, in g) and CPUE_n_ (B) of the different groups of fish, shrimp and squid species per seasons.** Fish species are grouped as planktivorous/piscivorous (PlankPisc), benthivorous/piscivorous (BenthPisc), or strictly benthivorous (StricBenth).

In the Sylt-Rømø Bight, a strong seasonal pattern was observed in the CPUEm with low values in winter (83 g.h^-1^) and much higher values in summer 411 g.h^-1^ (Fig 2A). In all seasons, the CPUE_m_ were largely dominated by planktivorous/piscivorous species in the Sylt-Rømø Bight (Fig 2A), ranging from 45 g.h^-1^ (54.2% of the total biomass (TB)) to 321 g.h^-1^ (78.2% of the TB). Second highest CPUE_m_ is represented by *Loligo spp*. in spring (27 g.h^-1^, 13.0% of the TB), and is equally spread between benthivorous/piscivorous and benthivorous species in summer (42 g.h^-1^, 10.0% of the TB and 30 g.h^-1^, 7.3% of the TB, respectively), fall (26 g.h^-1^, 15.1% of the TB and 31 g.h^-1^, 18% of the TB, respectively) and winter (19 g.h^-1^, 22.9% of the TB and 15 g.h^-1^, 18.3% of the TB, respectively). The proportion of *O*. *eperlanus* CPUE_m_ increased in summer compared to other seasons, but remained still low (15 g.h^-1^, 4% of the TB; Fig 2A).

The highest CPUE_n_ were recorded for planktivorous/piscivorous in spring (50 ind.h^-1^) and summer (139 ind.h^-1^; Fig 2B) whereas benthivorous species were the most abundant in fall (43 ind.h^-1^) and winter (14 ind.h^-1^). The second most abundant groups were benthivorous species in spring and summer, and planktivorous/piscivorous species in fall (Fig 2B). In winter, the second most abundant groups were both planktivorous/piscivorous and benthivorous/piscivorous species in equivalent importance (Fig 2B).

### Stable isotope composition of prey species

#### Sylt-Rømø Bight

**Fig 3:**
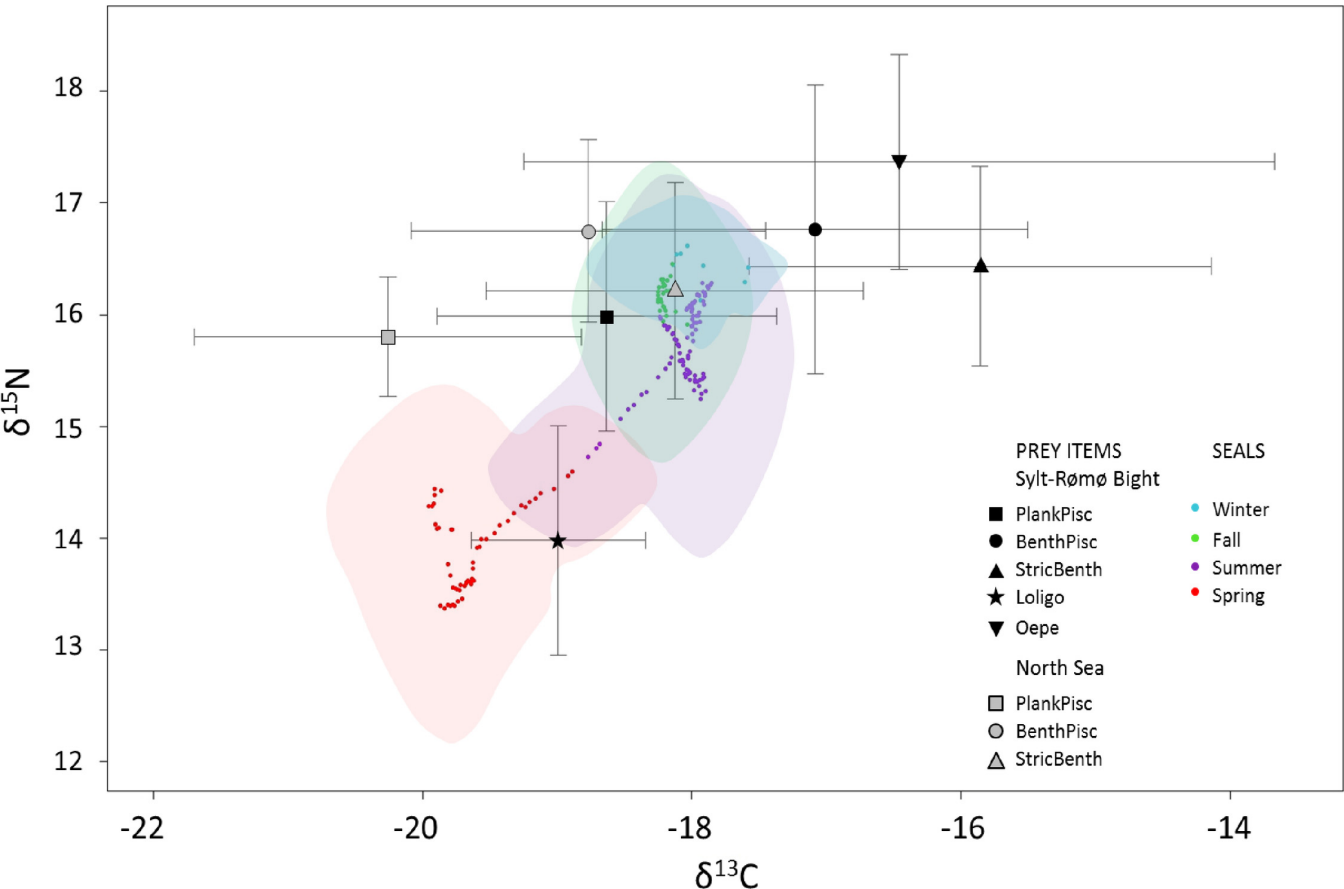
Mean stable isotope compositions of the groups of prey items (error bars show standard deviations) compared to the moving mean of seal vibrissae per season. The shaded areas represent the isotopic range per season including all standard deviations from each value of the moving mean. Theoretical stable isotope values of prey foraged by seals were computed with TFFs of 3.2 ‰ and 2.8 ‰ for δ^13^C and δ^15^N, respectively. Fish species are grouped as planktivorous/piscivorous (PlankPisc), benthivorous/piscivorous (BenthPisc), strictly benthivorous (StricBenth).

In the Sylt-Rømø Bight, δ^13^C values of potential prey items ranged from -23.5‰ (*O*. *eperlanus*) to -11.1‰ (*P*. *platessa*; S2 Table). On a yearly basis, planktivorous/piscivorous species and *Loligo spp*. were significantly more ^13^C-depleted than *O*. *eperlanus*, benthivorous/piscivorous, benthivorous species, and benthivorous species were more ^13^C-enriched than benthivorous/piscivorous species (Tables 3 and 4, Fig 3). δ^15^N values of potential prey items ranged from 12.2‰ (*C*. *harengus*) to 21.1‰ (*M*. *merlangus*). The benthivorous/piscivorous species and *O*. *eperlanus* had the highest δ^15^N values, followed in decreasing order by benthivorous species, planktivorous/piscivorous species and *Loligo sp* (Tables 3 and 4, Fig 3). The five groups of prey items (planktivorous/piscivorous, benthivorous/piscivorous, benthivorous, *Loligo*, and *O*. *eperlanus*) were then well differentiated by their δ^13^C and/or δ^15^N values in the Sylt-Rømø Bight (Tables 3 and 4, Fig 3).

**Table 3:** δ^13^C and δ^15^N values (mean ± standard deviation) of the different groups of prey items in the Sylt-Rømø Bight and the North Sea. n: sample size.

	Planktivorous/piscivorous	Benthivorous/piscivorous	Benthivorous	Loligo spp.	Osmerus eperlanus
**δ**^**13**^**C**	** **	** **	** **	** **	** **
**Sylt-Rømø Bight**	-18.6 ± 1.3 ‰	-17.1 ± 1.6 ‰	-15.9 ± 1.7 ‰	-19.0 ± 0.7 ‰	-16.5 ± 2.8 ‰
	n = 141	n = 118	n = 177	n = 15	n = 20
**North Sea**	-20.3 ± 1.4 ‰	-18.8 ± 1.3 ‰	-18.1 ± 1.4 ‰	-	-
	n = 5	n = 23	n = 33		
**δ**^**15**^**N**					
**Sylt-Rømø Bight**	16.0 ± 1.0 ‰	16.8 ± 1.3 ‰	16.4 ± 0.9 ‰	14.0 ± 1.0 ‰	17.4 ± 1.0 ‰
	n = 141	n = 118	n = 177	n = 15	n = 20
**North Sea**	15.8 ± 0.5 ‰	16.8 ± 0.8 ‰	16.2 ± 1.0 ‰	-	-
	n = 5	n = 23	n = 33		

**Table 4:** Summary of Tukey tests following ANOVAs (for the Sylt-Rømø Bight) and Wilcoxon rank sum tests following Kruskal Wallis tests (for the North Sea) between the different groups of prey items. Fish species are grouped as planktivorous/piscivorous (PlankPisc), benthivorous/piscivorous (BenthPisc), strictly benthivorous (StricBenth).

δ^13^C						δ^15^N					
Sylt-Rømø Bight			North Sea			Sylt-Rømø Bight			North Sea		
p-value		Comparisons of means	p-value		Comparisons of means	p-value		Comparisons of means	p-value		Comparisons of means
***	< 0.001	PlankPisc < BenthPisc	**	0.008	PlankPisc < StricBenth	***	< 0.001	PlankPisc < BenthPisc	*	0.047	PlankPisc < BenthPisc
***	< 0.001	PlankPisc < StricBenth	°	0.077	PlankPisc < BenthPisc	***	< 0.001	*Loligo spp*. < BenthPisc	°	0.072	StricBenth < BenthPisc
***	< 0.001	PlankPisc < *O*. *eperlanus*	°	0.086	BenthPisc < StricBenth	°	0.069	StricBenth < BenthPisc			
***	< 0.001	*Loligo spp*. < BenthPisc				***	< 0.001	PlankPisc < *O*. *eperlanus*			
***	< 0.001	*Loligo spp*. < StricBenth				***	< 0.001	*Loligo spp*. < *O*. *eperlanus*			
***	< 0.001	*Loligo spp*. < *O*. *eperlanus*				**	0.002	StrictBenth < *O*. *eperlanus*			
***	< 0.001	BenthPisc < StricBenth				**	0.002	PlankPisc < StricBenth			
						***	< 0.001	*Loligo spp*. < StrictBenth			
						***	< 0.001	*Loligo spp*. < PlankPisc			

*** α risk < 0.001

** α risk < 0.01

* α risk < 0.05

° α risk < 0.10

#### North Sea

In the North Sea, the δ^13^C values of the prey items ranged from -22.6 ‰ (*Sprattus sprattus*) to -14.7 ‰ (*Crangon crangon*; S2 Table). On a yearly basis, planktivorous/piscivorous species were the most ^13^C-depleted followed in increasing order by benthivorous/piscivorous species and benthivorous species (Tables 3 and 4, Fig 3). The δ^15^N values ranged from 13.7 ‰ (*P*. *platessa*) to 18.0 ‰ (*Ciliata mustela*). The benthivorous/piscivorous species were more enriched in ^15^N compared to the planktivorous/piscivorous and benthivorous species (Tables 3 and 4, Fig 3). As a result, as in the Sylt-Rømø Bight, the three groups of prey items from the North Sea (planktivorous/piscivorous, benthivorous/piscivorous and benthivorous) are well differentiated owing to their isotopic compositions (Fig 3).

#### Comparison of prey species between the Sylt-Rømø Bight and North Sea

The comparison between stable isotope composition of prey items in the Sylt-Rømø Bight and the North Sea revealed that prey items from each trophic group were significantly more ^13^C-depleted in the North Sea than in the Sylt-Rømø Bight (Wilcoxon rank sum tests, p-values: Planktivorous/piscivorous: 0.012, benthivorous/piscivorous: < 0.001, benthivorous: < 0.001; Fig 3). However, the planktivorous/piscivorous group in the Sylt-Rømø Bight had similar stable isotopic composition to the benthivorous and benthivorous/piscivorous groups in the North Sea. Between the Sylt-Rømø Bight and North Sea, no difference of δ^15^N values was observed for groups of prey (Wilcoxon rank sum tests, p-values: planktivorous/piscivorous: 0.576, benthivorous/piscivorous: 0.799, benthivorous: 0.383; Fig 3).

### Stable isotopic composition of vibrissae

On a seasonal basis, the vibrissae were significantly more ^13^C-depleted in spring (-16.1 ± 0.4 ‰, n = 3) than in winter (-14.8 ± 0.5 ‰, n = 9), fall (-15.0 ± 0.6 ‰, n = 16) and summer (-14.7 ± 0.6 ‰, n = 5; Wilcoxon sum rank tests, all p-values < 0.001). The mean δ^15^N value of vibrissae was equal to 18.7 ± 1.1 ‰. For δ^15^N values the same seasonal trend was observed as for δ^13^C, with significantly lower δ^15^N values in vibrissae in spring (16.7 ± 1.2 ‰) than in winter (19.2 ± 0.4 ‰), fall (19.1 ± 0.9 ‰) and summer (19.0 ± 0.6 ‰; Wilcoxon rank sum tests, all p-values < 0.001).

### Seasonal variation of the harbor seal´s diet

In every season, the δ^13^C values of the theoretical prey items were calculated by subtracting the trophic enrichment factor from the vibrissae values and ranged between those of the prey items from the North Sea and the Sylt-Rømø Bight (Fig 3). In spring, δ^15^N values of theoretical prey items were much lower than in other seasons and were close to those of the *Loligo* group (Fig 3).

**Fig 4:**
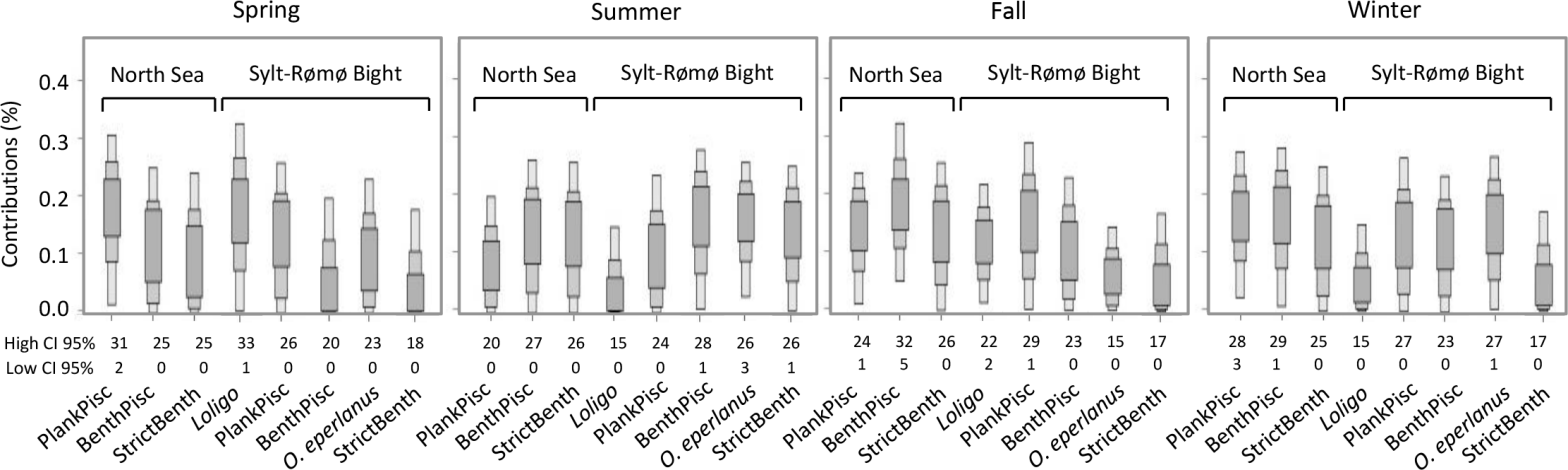
Contributions per season of the different trophic groups of prey items to diet of seals. Contributions were computed by the SIAR mixing model. Higher and lower values of the 95% credibility intervals (CI) are shown for each trophic group and each season. Fish species are grouped as planktivorous/piscivorous (PlankPisc), benthivorous/piscivorous (BenthPisc) or strictly benthivorous (StricBenth).

At both locations (i.e., Sylt-Rømø Bight and North Sea), planktivorous/piscivorous had a high contribution to the diet in spring (CI 95 from 0% to 26% and from 2% to 31%; Fig 4). In the Sylt-Rømø Bight *Loligo spp*. had the highest contribution to the diet in spring (CI 95 from 1% to 31%). In summer, benthivorous/piscivorous species (CI 95 from 1% to 28% in the Sylt-Rømø Bight and from 0% to 27% in the North Sea) and benthivorous species (C I95 from 1% to 26% in the Sylt-Rømø Bight and from 0% to 26% in the North Sea) dominated the diet. *O*. *eperlanus* had the second highest contribution in the Sylt-Rømø Bight in summer (CI 95 from 3% to 26%; Fig 4).

In fall and winter, the order of contribution of the group of prey items from the Sylt-Rømø Bight differed from the contribution of these groups from the North Sea. In the Sylt-Rømø Bight, planktivorous/piscivorous had the highest contribution in fall (CI 95 from 1% to 29%) and in winter (CI 95 from 0% to 27%) together with *O*. *eperlanus* (from 1% to 27%). In the North Sea, planktivorous/piscivorous, benthivorous/piscivorous and benthivorous had a relatively high contribution with a dominance of benthivorous/piscivorous in fall (CI 95 from 5% to 32%) and a dominance of benthivorous/piscivorous (CI 95 from 1% to 29%) and planktivorous/piscivorous (CI 95 from 3% to 28%) in winter (Fig 4).

## Discussion

### Large seasonal variation of prey species availability in the Sylt-Rømø Bight

Fish abundance observed in the Sylt-Rømø Bight was dominated by planktivorous/piscivorous and benthivorous species followed by benthivorous/piscivorous species. Biomass was also dominated by planktivorous/piscivorous species in the Sylt- Rømø Bight, mostly *C*. *harengus* and *A*. *tobianus*.

The seasonal patterns of biomass and abundance of fish observed in the Sylt-Rømø Bight are in accordance with the life cycle of several species, as already observed in the Wadden Sea and North Sea by other authors [70–73]. In the Sylt-Rømø Bight, biomass and abundance of fish are at their maximum in summer and minimum in winter. This temporal pattern is caused by two main reasons. First, the Wadden Sea is an important nursery area for juveniles of several fish species from the North Sea such as *C*. *harengus*, *M*. *merlangus* and *L*. *limanda* colonizing the tidal inlets and tidal flats in summer [70–73]. Second, in addition to juveniles, seasonally migrating species are found in the Wadden Sea. Most of these non-resident species migrate into the coastal zone in spring and leave in fall, when they go to deeper waters in the North Sea [72]. As a result, the Sylt-Rømø Bight can provide a much higher amount of food resources to seals in summer than in winter.

Some seasonal patterns are observed between the different trophic groups of prey species, which affect their availability to seals. Indeed, planktivorous/piscivorous species (e.g., *C*. *harengus* and *A*. *tobianus*) dominate the biomass in the Sylt-Rømø Bight but their abundance is high only in spring and summer. This indicates the presence of small sized individuals in spring and summer in contrast to relatively large individuals in fall and particularly in winter. This observation is in accordance with the high abundance of post larvae of *C*. *harengus* found in April and May by Dickey-Collas, Bolle [74] in the German Bight.

Benthivorous species have the highest abundance in fall and winter, which is mainly due to the high amount of *C*. *crangon* in these seasons (88% and 80% of the biomass of benthivorous species, respectively). The biomass and abundance of benthivorous species increased in summer in the Sylt-Rømø Bight. This is related to: (1) the recruitment period of *P*. *minutus* and *P*.*microps* [75] and (2) the settlement of *P*. *platessa* juveniles in April [76], following offshore spawning in January and February [70].

The abundance of benthivorous/piscivorous species (e.g., *M*. *merlangus*, *L*. *limanda*) decreased from summer to fall while the total biomass remained stable. This indicates the presence of juveniles from benthivorous/piscivorous species in summer in accordance to the spawning period of *M*. *merlangus* and *L*. *limanda* from February to May [70]. The highest biomass of *M*. *scorpius* was observed in winter. This might be explained by the spawning from December to February when the adults are mobile and are therefore more easily caught with a trawl net [77]. *G*. *morhua* had also its highest biomass in winter which corresponds to the concentration of the juveniles in shallow water during their first winter, as observed along the coasts of Denmark, Germany and the Netherlands [78].

Little is known about the seasonal distribution of *Loligo spp*. in the Wadden Sea, but the biomass peak observed in the Sylt-Rømø Bight in spring is in accordance with their seasonal migration from the English Channel to the North Sea [76].

### Coastal vs. offshore gradient in stable isotope composition of prey items

In the Sylt-Rømø Bight, prey species showed a classical gradient of ^13^C-enrichment, from planktivorous/piscivorous species (-18.6‰) and *Loligo spp*. (-19.0‰)—revealing, by their low δ^13^C values, an influence of pelagic food resources [79]—to benthivorous/piscivorous (-17.1‰) and strictly benthivorous (-15.9‰) species being more influenced by benthic food resources (Table 3). This gradient is related to the ^13^C-depletion of planktonic compared to benthic algae [80, 81]. The presence of small sized pelagic fish and cephalopods in the diet of *M*. *merlangus* [70, 82], the main benthivorous/piscivorous species, might explain the lower δ^13^C values of this group in comparison with benthivorous species (e.g., *Pomatoschistus spp*., *P*. *platessa* and *Zoarces viviparus*), feeding only on benthic macrofauna [75, 83].

The δ^15^N values of the prey species in the Sylt-Rømø Bight encompassed a large range, from 12.2 to 21.1‰, demonstrating that the considered species covered several trophic levels [37]. The low δ^15^N values of *Loligo spp*. (14.0‰) suggest that these prey species have a lower trophic level than the other groups of potential prey items (from 16.0‰ to 17.4‰), which is in contrast to stomach content observations showing that *Loligo spp*. prey on fish, crustacean, polychaetes and other cephalopods [79]. However, the δ^15^N values calculated for squids from the Atlantic Ocean (11.31 ± 2.06‰) and from temperate coastal and shelf areas (11.1 ± 2.1‰) by [84], and measured in *Loligo spp*. from the North Sea (12.9‰) [85] are even lower than those from the Sylt-Rømø Bight (14.0‰). These low δ^15^N values suggest that trophic enrichment factors in *Loligo spp*. are lower than those in fish, maybe due to different metabolic processes.

In the North Sea, the same benthic *vs*. pelagic gradient was observed for the δ^13^C values of benthivorous, benthivorous/piscivorous and planktivorous/piscivorous species as in the Sylt-Rømø Bight and can be explained in a similar way. Although the difference between planktivorous/piscivorous and benthivorous groups was not significant, the δ^15^N values followed the same trend as in the Sylt-Rømø Bight, with a ^15^N-enrichment from planktivorous/piscivorous to benthivorous and to benthivorous/piscivorous.

On a spatial scale, an inshore-offshore pattern was observed between the prey items in the Sylt-Rømø Bight and the ones in the North Sea. The prey species from the North Sea were predominantly influenced by oceanic food resources, while prey species in the Sylt-Rømø Bight were strongly influenced by benthic food resources [36, 86, 87]. A similar inshore-offshore gradient of δ^13^C values was observed by Le Loc'h, Hily (87) in the Bay of Biscay.

### Influence of pelagic prey species to the seal’s diet in spring compared to summer

Temporal variations of δ^13^C values indicate a shift from a diet more strongly influenced by pelagic prey items in spring to a diet of more benthic prey items in summer [80, 81]. This change is observed in both locations, the Sylt-Rømø Bight and the North Sea. In spring, the much lower δ^15^N values of seals are close to those of *Loligo spp*. As a result, it is very likely that seals forage more intensely on *Loligo spp*. during this season. In spring and summer, a smaller number of individuals were included in the data analysis compared to fall and winter. Indeed, the young-of-the-year were not old enough to forage throughout the year, and their stable isotope composition, which was influenced by lactation and weaning periods, was not included in the data analysis in spring and summer.

Nevertheless, the seasonal variation in the harbor seal’s diet observed in spring and summer is in accordance with studies by Brown and Pierce (21), Hall, Watkins (22), Andersen, Teilmann (28) and Berg, Haug (88) conducted in the southern North Sea. They show a high occurrence of pelagic species in spring (e.g., *C*. *harengus* and *A*. *tobianus*) and an increase of gadoids (e.g., *M*. *merlangus*) and flat fish (e.g., *P*. *platessa*, *Solea solea*, *P*. *flesus*) in seals gut contents in summer. This shift can be explained by a change in the availability of fish species [21, 25, 88]. In the Sylt-Rømø Bight, the high contribution of planktivorous/piscivorous and *Loligo* species to seals diet in spring coincides with the highest contribution of these two groups to the fish biomass in the Sylt-Rømø Bight, particularly the seasonal peak of *Loligo spp*.

### Harbor seals as benthic feeders

Although the biomass and abundance of planktivorous/piscivorous species remain very high in summer, highest contribution of benthivorous species to seals diet are observed in this season, when biomass and abundance of these species show their maximum in the Sylt-Rømø Bight. This confirms the opportunistic behavior of harbor seals foraging on one of the most abundant prey species in the sea, but not necessarily on the most abundant one [25]. Furthermore, the higher consumption by seals of benthivorous/piscivorous and benthivorous species when they become more available in summer confirms that harbor seals are primarily benthic feeders [6]. This observation is supported by the results of gut content analysis conducted in the Wadden Sea in Schleswig Holstein, in which flat-fish (benthivorous/piscivorous and benthivorous) and gadoids (benthivorous/piscivorous) were observed as main prey items [17, 18, 27]. Furthermore, Härkönen (20) showed that along the Danish coast of the North Sea, harbor seals consume the most abundant gadoid (benthivorous/piscivorous) species but do not feed on several other species of fish that are also numerous in this area.

### Higher use of the North Sea resources in fall and winter

In fall and winter, outputs of the SIAR mixing models describe that harbor seals have a diet mostly based on pelagic species in the Sylt-Rømø Bight and/or on benthic species in the North Sea. The very low biomass observed in the Sylt-Rømø Bight during these seasons particularly in winter, suggests that the contributions of Sylt-Rømø Bight food sources were overestimated by the SIAR models. Furthermore, gut content studies of North Sea harbor seals found gadoids (e.g., *M*. *merlangus*, *G*. *morhua*) as main prey items in fall and winter [21, 22, 28, 88]. This is in accordance with the high contribution of benthivorous/piscivorous from the North Sea in fall and winter (5% to 32% and 1% to 29% respectively). Harbor seals might forage more in the North Sea than in the Sylt-Rømø Bight in these seasons. This hypothesis is supported by telemetry studies showing that seals tagged on the Rømø Island show strong seasonal variations in foraging behavior, with significantly longer foraging trips to the North Sea in winter independently of the age or the sex of the animals [6]. Furthermore, Jensen (54) counted in the Sylt-Rømø Bight about 80% less adult seals in December than in August. This decrease of seal abundance in the bight in winter support the hypothesis that seals might use more of the North Sea food resources in this season. A better knowledge about the stable isotope compositions of prey items from the North Sea and their seasonal and spatial variations would give a better understanding of foraging behavior of seals in the North Sea.

In summary, harbor seals might use the food resources of the Sylt-Rømø Bight and the North Sea in similar amounts in spring and summer with a shift from a pelagic based diet in spring to a benthic based diet in summer in both locations, whereas in fall and winter they probably forage more in the North Sea, seemingly on benthic influenced species.

## Conclusions

In this study, we observed resource changes and spatial changes. Indeed, a higher influence of pelagic food resources is evident in the harbor seal’s diet in spring whereas the diet is dominated by benthic food resources in summer, fall and winter. Furthermore, harbor seals might use more food resources of the Sylt-Rømø Bight in spring and summer compared to fall and winter when the biomass of prey items is relatively low. Thus, the Sylt-Rømø Bight has an important role as a foraging area for harbor seals in addition to its function as a resting and nursery area. The use of the Bight as a foraging area by a large colony of harbor seals might have a seasonal and relatively strong influence on the food web of the Sylt-Rømø Bight, particularly in spring and summer, when the seal abundance and the contribution of Sylt-Rømø Bight food resources to their diet are highest.

These results also highlight the necessity of much more detailed studies about temporal and spatial variations of marine mammal diets. For example, a potential competition of seals with fisheries for commercial species might strongly depend on seasons and location. Vibrissae can be used as very good recorders in marine mammal trophodynamics. Therefore, additional studies on growth rate of vibrissae are needed to precise the correspondence between the vibrissae length and the time scale. Furthermore, the combination of diet studies based on trophic markers such as stable isotopes with telemetry survey would be very valuable for management issues about highly mobile species such as harbor seals.

## Supporting Information

S1 Fig δ^13^C (filled circles and triangles) and δ^15^N (open circles and triangles) values along the length of two vibrissae from an adult (circles) and two vibrissae from a yearling (triangles). The total length (mm) of each vibrissa is expressed in the legend in between parentheses. Information about both individuals is displayed in Table 1.
(TIF)

S2 Fig Reconstruction of temporal variation of carbon isotopic composition along vibrissae of two adults (seals ID 1 and ID 19; Table 1) and two young-of-the-year (seals ID 11 and ID 18; Table 1). (PDF)

S1 Table mean values and standard deviations of harbor seals adults and young-of-the-year, difference between the mean value of young-of-the-year and the mean value of adults (Δδ^15^N and Δδ^13^C), results of the non-parametric Wilcoxon tests comparing the values of young-of-the-year and adults per month for δ^15^N and δ^13^C. Data in bold, corresponding to vibrissae from adults and section of vibrissae from young-of-the-year from and after September (older than 3–4 months), were used in this study.
(PDF)

S2 Table δ^13^C and δ^15^N values (mean ± standard deviation) of the prey items species from the Sylt-Rømø Bight and the North Sea. n: sample size. These data were collected from April 2008 to November 2009 [56] and from January to November 2013 (present study).
(PDF)
