# Supplementary figures and images for: Seasonal Variation of Harbor Seal's Diet from the Wadden Sea in Relation to Prey Availability
Source: PLoS One. 2016 May 13;11(5):e0155727. doi: 10.1371/journal.pone.0155727 (PMC4866785; doi:10.1371/journal.pone.0155727)

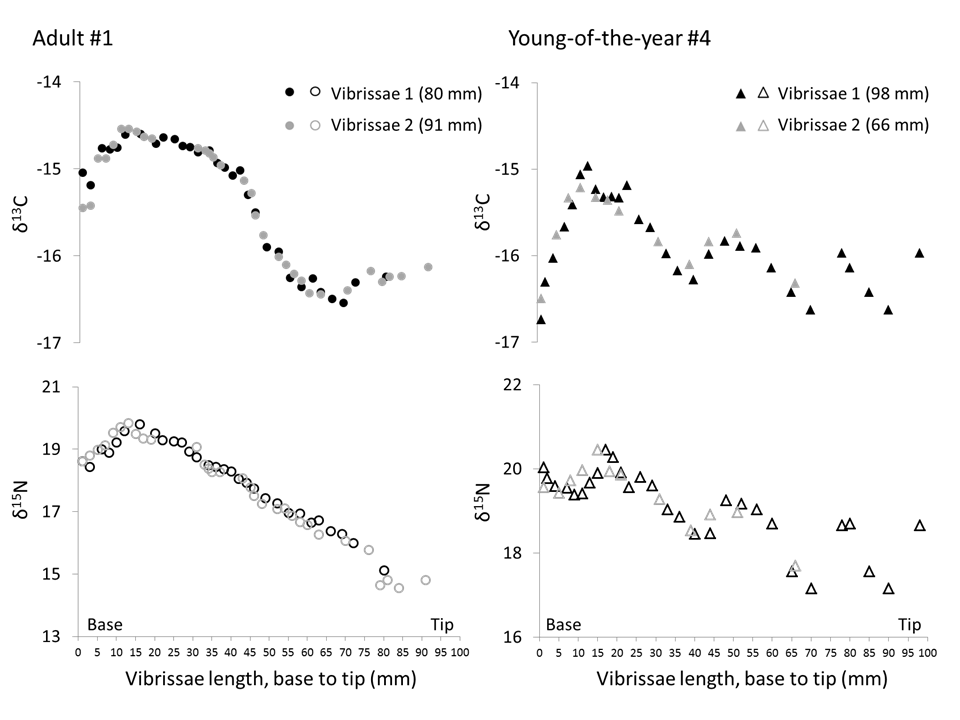

Supplement: S1 Fig — The total length (mm) of each vibrissa is expressed in the legend in between parentheses. Information about both individuals is displayed in Table 1. (TIF) [file pone.0155727.s001.tif]
