# Supplementary material for: Seasonal Variation of Harbor Seal's Diet from the Wadden Sea in Relation to Prey Availability
Source: PLoS One. 2016 May 13;11(5):e0155727. doi: 10.1371/journal.pone.0155727 (PMC4866785; doi:10.1371/journal.pone.0155727)
Supplement: S2 Fig — (PDF) [file pone.0155727.s002.pdf]

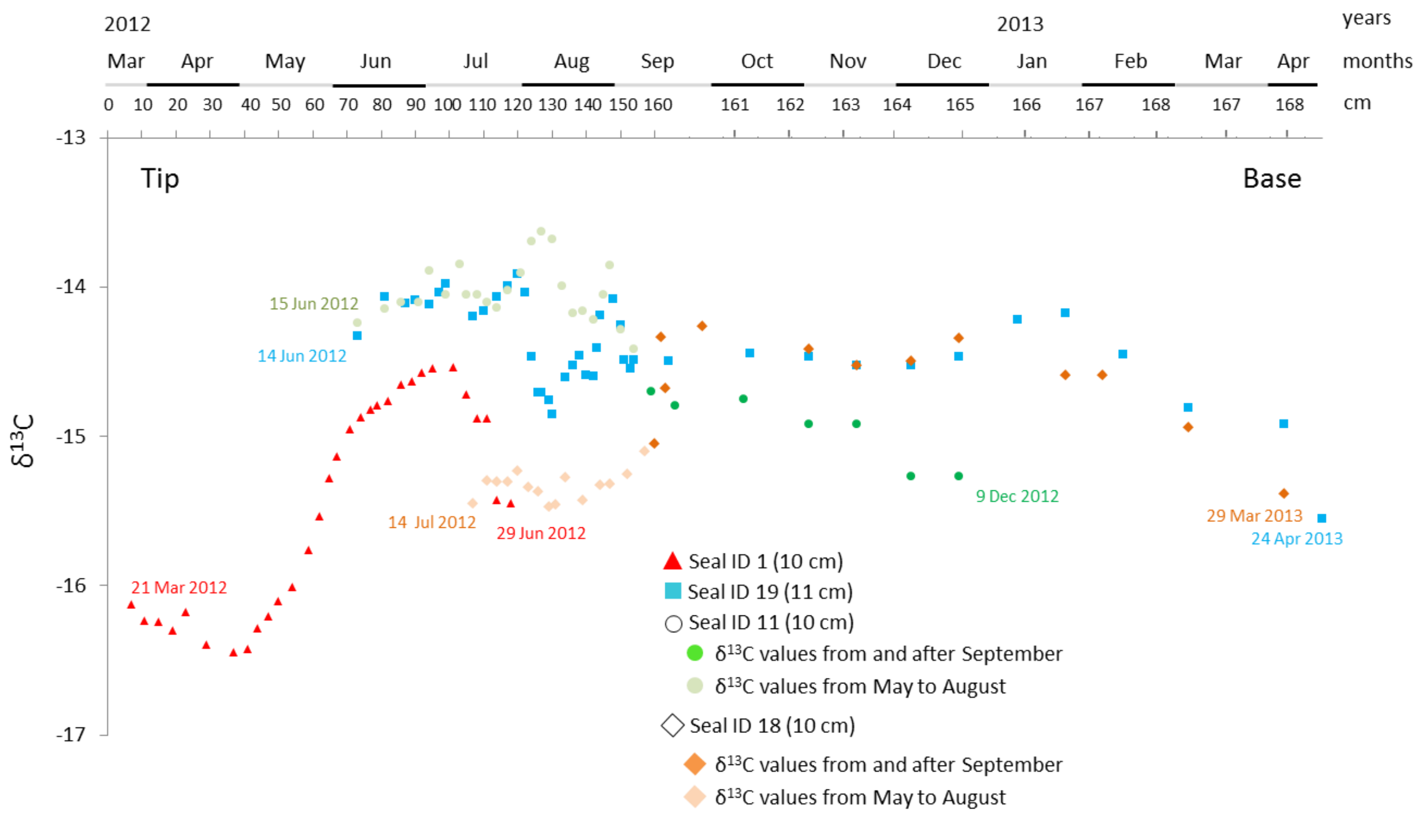

All vibrissae were treated in this manner for both Carbon and Nitrogen stable isotope ratios and were included in the moving mean for seasonal variation study.

Correspondence Time/Length: The growth rates used for reconstitution of temporal variation were 0.78 $mm.d^{-1}$ from May to September, and 0.075 $mm.d^{-1}$ from October to April [45].

Young-of-the-year's data treatment: the $\delta^{13}C$ and $\delta^{15}N$ values of young-of-the-year corresponding to May, June, July and August were not taking in account in the analyses for this study (S1 Table).
