# Supplementary material for: Seasonal Variation of Harbor Seal's Diet from the Wadden Sea in Relation to Prey Availability
Source: PLoS One. 2016 May 13;11(5):e0155727. doi: 10.1371/journal.pone.0155727 (PMC4866785; doi:10.1371/journal.pone.0155727)
Supplement: S1 Table — Data in bold, corresponding to vibrissae from adults and section of vibrissae from young-of-the-year from and after September (older than 3–4 months), were used in this study. (PDF) [file pone.0155727.s003.pdf]

Months	May	June	July	August	September	October	November	December
Corresponding age of young-of-the-year (months)	<1	0-1	1-2	2-3	3-4	4-5	5-6	6-7
**$\delta^{15}N$**								
Adults	**16.0 ± 0.1**	**18.0 ± 1.1**	**18.9 ± 1.4**	**19.8 ± 1.0**	**19.1 ± 0.8**	**19.0**	**19.0**	**19.4 ± 0.1**
Young-of-the-year	19.8 ± 0.8	19.7 ± 0.8	19.9 ± 0.8	20.0 ± 0.9	**19.3 ± 1.0**	**18.8 ± 0.9**	**19.3 ± 0.6**	**19.2 ± 0.4**
$\Delta\delta^{15}N$	3.8	1.7	1.0	0.2	0.2	0.3	0.3	0.2
Wilcoxon test p-value	0.009 **	0.048 *	0.061 º	0.332	0.574	-	-	0.801
**$\delta^{13}C$**								
Adults	**-16.6 ± 0.2**	**-15.2 ± 1.2**	**-15.0 ± 1.1**	**-14.4 ± 0.2**	**-14.9 ± 0.5**	**-14.5**	**-14.5**	**-14.2 ± 0.2**
Young-of-the-year	-14.9 ± 0.6	-15.1 ± 0.6	-15.0 ± 0.7	-14.8 ± 0.6	**-15.0 ± 0.7**	**-14.9 ± 0.6**	**-15.0 ± 0.6**	**-15.1 ± 0.5**
$\Delta\delta^{13}C$	1.7	0.1	0.0	0.4	0.1	0.4	0.5	0.8
Wilcoxon test p-value	0.009***	0.978	0.71	0.551	0.813	-	-	0.133

** α risk < 0.01   * α risk < 0.05   º α risk < 0.10
