# Supplementary material for: Seasonal Variation of Harbor Seal's Diet from the Wadden Sea in Relation to Prey Availability
Source: PLoS One. 2016 May 13;11(5):e0155727. doi: 10.1371/journal.pone.0155727 (PMC4866785; doi:10.1371/journal.pone.0155727)
Supplement: S2 Table — n: sample size. These data were collected from April 2008 to November 2009 [56] and from January to November 2013 (present study). (PDF) [file pone.0155727.s004.pdf]

Sylt-Rømø Bight

trophic group	species	d13C (‰)	d15N (‰)	n
planktivorous/ piscivorous	*Ammodytes tobianus*	-19.0 ± 1.2	16.0 ± 0.9	38
	Belone belone	-17.0 ± 1.1	16.5 ± 0.6	7
	Clupea harengus	-18.8 ± 0.4	16.0 ± 0.7	54
	Hyperoplus lanceolatus	-18.4 ± 1.6	16.0 ± 1.3	14
	Sprattus sprattus	-18.3 ± 1.9	15.8 ± 1.1	28
Benthivorous/ piscivorous	*Gasterosteus aculeatus*	-18.9 ± 1.4	16.9 ± 0.6	9
	Limanda limanda	-16.9 ± 0.6	16.6± 1.0	18
	Merlangius merlangus	-17.1 ± 1.9	16.8 ± 1.3	36
	Myoxocephalus scorpius	-15.6 ± 0.6	17.1 ± 1.0	17
	Platichthys flesus	-16.2 ± 1.3	16.2 ± 1.7	8
	Syngnathus rostellatus	-17.6 ± 1.0	16.8 ± 0.6	30
Strictly benthivorous	*Agonus cataphractus*	-15.9 ± 1.6	17.2 ± 0.9	19
	Crangon crangon	-16.1 ± 1.2	16.1 ± 1.3	9
	Pholis gunnellus	-16.6 ± 2.8	17.0 ± 1.0	9
	Pomatoschistus microps	-14.1 ± 1.6	17.0 ± 1.0	21
	Pomatoschistus minutus	-16.9 ± 1.0	16.5 ± 0.4	42
	Pleuronectes platessa	-15.7 ± 1.8	15.8 ± 0.5	61
	Zoarces viviparus	-15.6 ± 1.6	16.7 ± 0.6	16
	loligo sp.	-19.0 ± 0.8	14.0 ± 1.0	15
	Osmerus eperlanus	-16.5 ± 0.9	17.4 ± 0.7	20

North Sea

trophic group	species	d13C (‰)	d15N (‰)	n
planktivorous/ piscivorous	*Hyperoplus lanceolatus*	-19.8 ± 0.9	16.2 ± 0.1	3
	Sprattus sprattus	-21.0 ± 2.2	15.2 ± 0.1	2
Benthivorous/ piscivorous	*Ciliata mustela*	-18.2 ± 0.4	17.6 ± 0.6	2
	Gadus morhua	-18.4 ± 0.5	17.7 ± 0.3	3
	Limanda limanda	-20.0 ± 1.0	15.6 ± 0.2	6
	Merlangius merlangus	-18.4 ± 1.3	16.9 ± 0.4	12
Strictly benthivorous	*Agonus cataphractus*	-16.8 ± 0.1	17.4 ± 0.2	3
	Crangon crangon	-17.3 ± 2.2	16.2 ± 0.7	6
	Pomatoschistus microps	-19.1	15.0	1
	Pomatoschistus minutus	-17.9 ± 0.2	16.3 ± 0.1	5
	Pleuronectes platessa	-19.9 ± 0.9	14.7 ± 0.7	6
	Solea solea	-18.5 ± 0.7	16.4 ± 0.8	3
	Zoarces viviparus	-17.8 ± 0.6	16.8 ± 0.5	9
